# Interactions of host miRNAs in the flavivirus 3´UTR genome: From bioinformatics predictions to practical approaches

**DOI:** 10.3389/fcimb.2022.976843

**Published:** 2022-10-13

**Authors:** Rodolfo Gamaliel Avila-Bonilla, Juan Santiago Salas-Benito

**Affiliations:** ^1^ Institute of Immunology and Infection Research, School of Biological Sciences, University of Edinburgh, Edinburgh, United Kingdom; ^2^ Laboratorio de Biomedicina Moleculart 3, Maestría en Ciencias en Biomedicina Molecular, Escuela Nacional de Medicina y Homeopatía, Instituto Politécnico Nacional, Mexico City, Mexico

**Keywords:** arthropod-borne viruses, MicroRNAs, vaccines, antivirals, non-translated regions

## Abstract

The genus Flavivirus of the *Flaviviridae* family includes important viruses, such as Dengue, Zika, West Nile, Japanese encephalitis, Murray Valley encephalitis, tick-borne encephalitis, Yellow fever, Saint Louis encephalitis, and Usutu viruses. They are transmitted by mosquitoes or ticks, and they can infect humans, causing fever, encephalitis, or haemorrhagic fever. The treatment resources for these diseases and the number of vaccines available are limited. It has been discovered that eukaryotic cells synthesize small RNA molecules that can bind specifically to sequences present in messenger RNAs to inhibit the translation process, thus regulating gene expression. These small RNAs have been named microRNAs, and they have an important impact on viral infections. In this review, we compiled the available information on miRNAs that can interact with the 3’ untranslated region (3’UTR) of the flavivirus genome, a conserved region that is important for viral replication and translation.

## Introduction

The viruses that belong to the *Flaviviridae* family are classified into four main genera: *Flavivirus*, *Pegivirus*, *Pestivirus* and *Hepacivirus* (Neufeldt et al., 2018). The *Pegivirus* genus includes viruses designated GB (for the initials of the surgeon whom this virus was isolate) A, C, and D (Stapleton et al., 2011). The members of the *Pestivirus* genus include bovine viral diarrhoea virus 1 (BVDV-1) or Pestivirus A, bovine viral diarrhoea virus 2 (BVDV-2) or Pestivirus B, classical swine fever virus (CSFV) or Pestivirus C, border disease virus (BDV) or Pestivirus D, and others designated as Pestiviruses E to K (Riitho et al., 2020). The representative member of the *Hepacivirus* genus is human hepatitis C virus (HCV), and this genus also includes GBV-B virus (Stapleton et al., 2011; Neufeldt et al., 2018). Finally, the *Flavivirus* genus includes a large group of viruses that are organized into four groups according to their mechanism of transmission: mosquito-borne flaviviruses (MBFV), transmitted by mosquitoes; tick-borne flaviviruses (TBFV), transmitted by ticks; insect-specific flaviviruses (ISFV), which circulate only among mosquitoes and other insects such as soldier flies and sandflies; and no-known-vector flaviviruses (NKFV), found only in rodents and bats (Moureau et al., 2010; Cook et al., 2013; Blitvich and Firth, 2015; Ochsenreiter et al., 2019; Colmant et al., 2022).

MBFV and TBFV are particularly important for their ability to produce a variety of illnesses in humans. MBFV are transmitted by *Culex* and *Aedes* mosquitoes and include viruses such as yellow fever virus (YFV), dengue virus (DENV), Zika virus (ZIKV), West Nile virus (WNV), Japanese encephalitis virus (JEV), Usutu virus (USUV), Saint Louis encephalitis virus (SLEV), and Murray Valley encephalitis virus (MVEV). TBFV includes tick-borne encephalitis virus (TBEV) (Ochsenreiter et al., 2019).

The disease caused by YFV is biphasic with viremic and toxaemic phases. The viremic phase is characterized by symptoms of fever, anorexia, myalgia, and headache. The toxaemic phase is potentially lethal, and the symptoms again include fever, myalgia, and headache, with the additional symptom of jaundice (Nóbrega Litvoc et al., 2018). DENV comprises four serotypes (DENV 1-4) with approximately 65% homology at the amino acid level. Dengue fever is usually a mild disease with symptoms of fever, myalgia, arthralgia, headache, severe retro-orbital pain, anorexia, nausea, vomiting, skin erythema, and conjunctivitis. However, some patients can evolve to a more severe outcome characterized by an increase in capillary permeability and coagulation dysfunction that leads to potentially lethal haemorrhagic manifestations (Harapan et al., 2020). The other MBFV and TBFV are causative agents of encephalitis. WNV, SLEV, and MVEV belong to the JEV serocomplex and are transmitted by *Culex* mosquitoes (Salimi et al., 2016; Habarugira et al., 2020). JEV and MVEV predominantly cause encephalitis in children, whereas WNV or SLEV mainly occur in immunocompromised adults (Salimi et al., 2016). WNV can infect a variety of animals, including birds, horses, sheep, reptiles, cats, and rodents; it can also infect humans, causing meningitis, encephalitis, and acute flaccid paralysis (Habarugira et al., 2020). JEV infects humans and occasionally animals. JEV and MVEV are responsible for an outcome characterized by high fever, headache, neck stiffness, disorientation, seizures, paralysis, coma, and eventual death (Salimi et al., 2016; Ashraf et al., 2021). SLEV occasionally causes encephalitis in the USA (Salimi et al., 2016). ZIKV is transmitted by *Aedes* mosquitoes, and most infections are asymptomatic and clinical manifestations include rash, fever, arthralgia, myalgia, and conjunctivitis in adults. The neurological manifestations include meningoencephalitis or Guillain–Barré syndrome. Infection in pregnant women can result in foetal malformations, such as microcephaly, structural brain abnormalities and ocular anomalies (Masmejan et al., 2020). Finally, TBEV include three groups: mammalian tick-borne flaviviruses (M-TBFV), seabird tick-borne flaviviruses (S-TBFV) and the Kadam virus group (Ochsenreiter et al., 2019). They can infect a wide range of animals, including humans; in humans, they can cause a mild or moderate febrile illness with fatigue, general malaise, headache, and muscular pain but can also cause illnesses ranging from mild meningitis to severe encephalitis (Yoshii, 2019).

## MicroRNAs

MicroRNAs (miRNAS) are small noncoding RNAs (approximately 22-25 nt) that control gene expression at the posttranscriptional level in eukaryotic cells (Berezikov, 2011; Dexheimer and Cochella, 2020). Two pathways of miRNA biogenesis have been characterized, the canonical and non-canonical. The canonical pathway begins with the transcription of a primary product called pri-miRNA performed by RNA polymerases II (pol II) (Lee et al., 2004) and III (pol III) (Borchert et al., 2006) in the nucleus, using miRNAs genes that are present in intergenic regions or organized in polycistronic clusters as a template (Rodriguez et al., 2004). Then, the microprocessor complex that contains the RNase III enzyme Drosha and DiGeorge Syndrome Critical Region 8 protein (DGCR8), recognizes the pri-miRNA and cleaves the hairpin structure to produce 5′-monophosphate and a 3′-2-nt overhang precursor miRNA (pre-miRNA) (Cullen, 2004; Gregory et al., 2004). The pre-miRNA is recognized and exported by exportin 5 (XPO5) and RAS-related nuclear protein-guanosine-5’-triphosphate-ase (Ran-GTPase) to the cytoplasm where the terminal loop is removed by the endonuclease Dicer to generate a short miRNA duplex (Okada et al., 2009). It should be noted that in mammalian cells pre-miRNAs and silencing RNAs (siRNAs) are processed by the same RNase enzyme III (Dicer), but in insects the processing of pre-miRNAs is conducted by Dicer-1, while RNase Dicer-2 is involved in the maturation pathway of siRNAs (Asgari, 2015).

Then, the miRNA duplex is loaded in the agonist protein (AGO) of the RNA-induced silencing complex (RISC) to promote miRNA-messenger RNA (mRNA) interactions, primarily in the 3´ untranslated region (3’UTR, canonical interaction) but also in the 5’UTR and open reading frames (ORFs) of the mRNAs to induce mRNA silencing and decay (Shabalina and Koonin, 2008; Eichhorn et al., 2014; Bartel, 2018) or increase the mRNA stability and translation (Fabian et al., 2010; Hussain et al., 2011; Zhang et al., 2014). In humans, there are four types of AGO (1-4), and all proteins are capable of inducing mRNA decay and repression by incorporating siRNA and miRNA duplexes. By contrast, invertebrates have two AGO (1-2) and they possess a strict RNAi-sorting system, AGO1 favors miRNAs bindings over AGO2, which prefers siRNAs (Ha and Kim, 2014).

The non-canonical miRNA pathway could be processed by several mechanisms in which other noncoding RNAs are involved, including the processing of endogenous introns, snoRNAs, tRNAs, and shRNAs that derivate to unconventional pre-miRNA (approximately 22 nucleotides). It has been reported that non-canonical miRNAs can be produced in a microprocessor-independent or Dicer-independent manner. An example is miRtrons biogenesis, where the pri-miRNAs sequence corresponds to the entire intronic gene, and after the splicing, the pre-miRNA is processed by Dicer to create a mature miRNA (Yang and Lai, 2011; Ha and Kim, 2014; Annese et al., 2020). In other cases, pri-miRNAs are directly loaded into AGO2 to catalyze the maturation of the microRNA in a Dicer-independent manner, this includes miR-451 (Cheloufi et al., 2010) and IsomiRs (Liang et al., 2017).

MiRNAs play important roles during flavivirus-host interactions (Kozak et al., 2017) and it has been reported that miRNA biogenesis proteins can directly interact with flavivirus replication machinery. After infection with DENV, human cells showed a reduction in the expression levels of Drosha, Dicer, DGCR8, and AGO proteins and the silencing of these genes in virus-infected cells increases DENV replication (Kakumani et al., 2013; Casseb et al., 2016). Furthermore, the DENV-NS4B protein is a crucial suppressor of the host miRNA pathway (Kakumani et al., 2013), and DENV-NS3 protein interacts with HSPA1A, which is associated with AGO proteins, and impact on viral suppression of the miRNA biogenesis proteins (Kakumani et al., 2020). In ZIKV infection, Dicer is the top binding protein for the capsid protein and this interaction mediates inhibition of Dicer and causes microcephaly phenotype in a mice model (Zeng et al., 2020).

In insect-flavivirus interactions, Dicer proteins appear to be equally important (Angleró-Rodríguez et al., 2017; Mukherjee et al., 2019; Harsh and Eleftherianos, 2020; Qiu et al., 2020). For instance, the inhibition of Dicer-2 results in the reduction of the Vago protein, which is an important player in the control of the infection by WNV (Paradkar et al., 2014). Also, the phenotypic resistance to DENV observed in some mosquito strains was associated with some Dicer 2 polymorphism (Lambrechts et al., 2013), and Dicer 2 mutations in fly models increases susceptibility to ZIKV infection (Harsh et al., 2018). Interesting, the 3’ UTR-derived subgenomic flavivirus RNA (sfRNA) efficiently suppressed the miRNA pathway in mammalian and insect cells promoting viral propagation (Schuessler et al., 2012; Moon et al., 2015; Bavia et al., 2016). It has been reported that sfRNA of WNV inhibits cleavage of double-stranded RNA by Dicer *in vitro* (Bavia et al., 2016).

## Flavivirus 3’ UTR

Flaviviruses have a single RNA strand genome of approximately 11 kb nucleotides in length with positive polarity. The genome has a single ORF that encodes a polyprotein that is processed by viral and cellular proteases to generate 3 structural and 7 nonstructural proteins (Harapan et al., 2020). The ORF is flanked by two UTRs: 5’ and 3’. The 3’UTR of flaviviruses replaced the poly(A) tail present in other viral and cellular mRNAs (Holden and Harris, 2004). It is important for viral translation and replication (Koraka et al., 2009; Wei et al., 2009; Berzal-Herranz et al., 2022) and is also associated with virulence (Koraka et al., 2009; Thaisonthi et al., 2013). This region is the target of several cellular proteins, such as translation elongation factor 1α (EF-1α); polypyrimidine tract binding protein (PTB); autoantigen La; p100; RNA binding motif protein, X-linked (RBMX); and insulin like growth factor 2 mRNA-binding protein 1 (IF2B1) (De Nova-Ocampo et al., 2002; García-Montalvo et al., 2004; Lei et al., 2011). It also has several putative sites for the interaction of Musashi proteins (Msi), a family of proteins that act as translational regulators of mRNA involved in cell proliferation and differentiation (Schneider and Wolfinger, 2019). Msi1 interacts with the ZIKV genome and enhances viral replication (Chavali et al., 2017).

**Figure 1:**
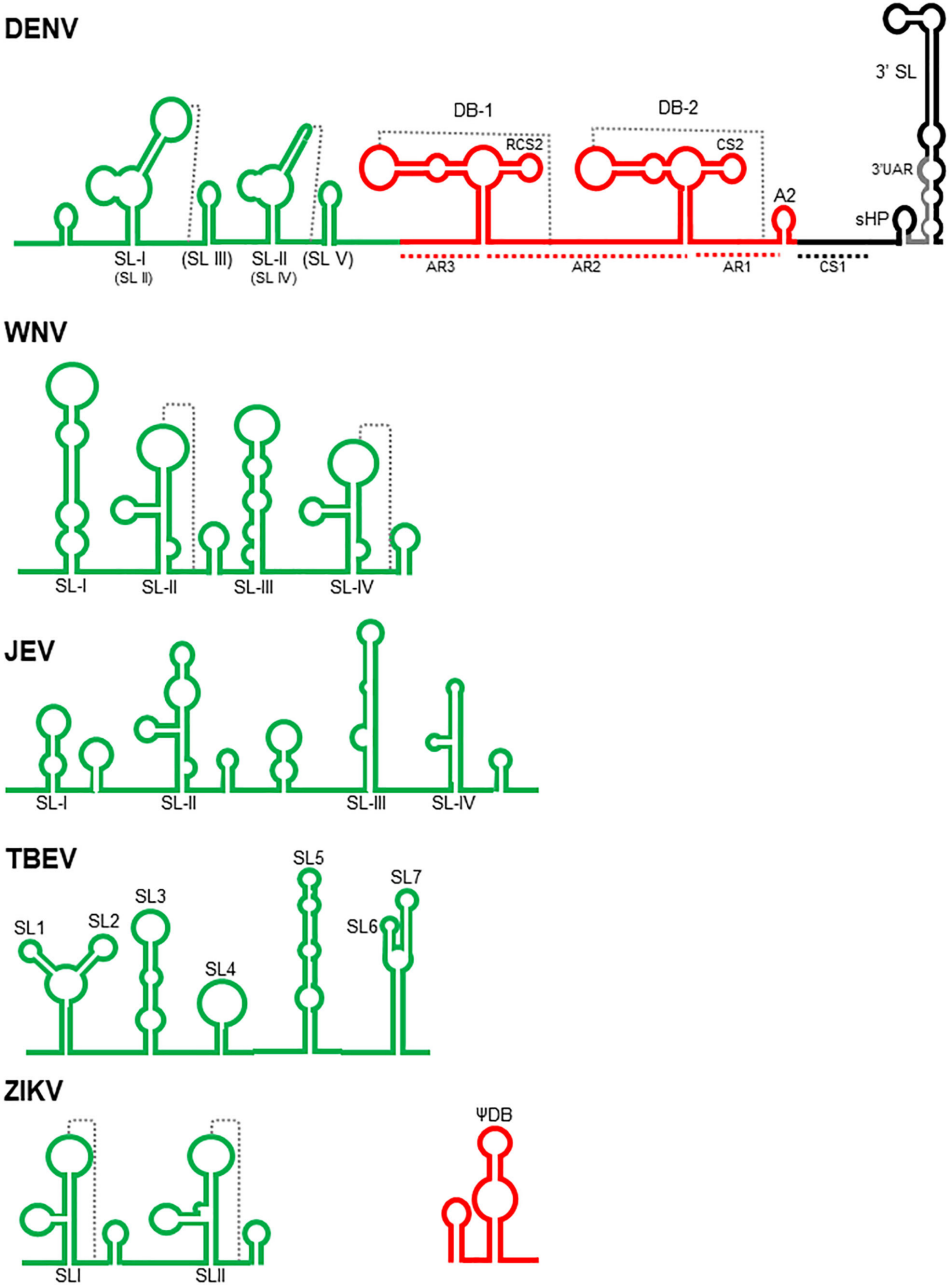
Schematic structure of the flavivirus 3’ UTR. The complete 3’ UTR of Dengue virus (DENV) is shown according with Olsthoorn and Bol, 2001; Romero et al., 2006; Blaney et al., 2008; Chapman et al., 2014; Kasprzak and Shapiro, 2014; Villordo et al., 2015; Filomatori et al., 2017; Liu et al., 2018; de Borba et al., 2019; Finol and Ooi, 2019, and Bellone et al., 2020. The variable, core and 3’ terminal regions are colored in green, red, and black, respectively. SL, stem loops; DB, dumbbells, sHP, small hairpin. The pseudoknots are indicated with dotted lines. The nomenclature referred by Liu et al., 2018 for the stem loops present in the variable region are in parenthesis. The variable regions of West Nile (WNV- Tilgner et al., 2005; Funk et al., 2010; Clarke et al., 2015, and Göertz et al., 2016), Japanese encephalitis (JEV- Chen et al., 2018 and Xing et al., 2021), Tick borne encephalitis (TBEV- Muto et al., 2018 and Sakai et al., 2015) and Zika viruses (ZIKV- Schneider and Wolfinger, 2019, and Sparks et al., 2020) are shown. Additionally, the first dumbbell structure of ZIKV (Schneider and Wolfinger, 2019) is also included.

The 3’UTR (**Figure 1**) is approximately 388-462 nucleotides long and encompasses three regions: a variable region (VR), located immediately downstream of the stop codon; a core region; and a 3’-terminal region (Proutski et al., 1997; Markoff, 2003; Romero et al., 2006; Zhou et al., 2006). In TBE viruses, only two regions are identified: the variable region and the core region (Sakai et al., 2015; Muto et al., 2018).

In the VR region, most insertions and deletions occur among the DENV isolates (Koraka et al., 2009; Liu et al., 2018; Mo et al., 2018; Muto et al., 2018); therefore, it has served as a good marker to analyse viral evolution (de Castro et al., 2013) and is associated with viral fitness (Villordo et al., 2015). It interacts with proteins related to stress granules (SGs) and processing bodies (P-bodies), such as Caprin1, G3BP1, G3BP2 and USP10 (Ward et al., 2011). In this region, several stem-loop structures with several designations have been identified according to the virus and the authors (**Figure 1**). SLII and SLIV in WNV (Funk et al., 2010; Göertz et al., 2016) and SLI and SLII in DENV (Chapman et al., 2014; Villordo et al., 2015) are able to form pseudoknots. In some TBE viruses, there are seven stem–loops: SL1-SL7 (Sakai et al., 2015; Ochsenreiter et al., 2019). These SL structures are conserved among flaviviruses and are responsible for the inhibition of the activity of Xrn-1 nuclease, an important process for the generation of sfRNAs, which participate in the antiviral evasion mechanisms in both mammals and mosquitoes (Funk et al., 2010; Villordo et al., 2015; Göertz et al., 2016; Chen et al., 2018; Bellone et al., 2020), including viral adaptation, fitness, virulence, and tissue tropism (Funk et al., 2010; Liu et al., 2018; Finol and Ooi, 2019; Sparks et al., 2020). In WNV, the SL-III has an inhibitory effect on translation since its deletion increase viral translation efficiency (Berzal-Herranz et al., 2022). In ZIKV, the SLI element has a putative binding site for the Msi protein, and the interaction of this protein with the viral genome has been proposed to inhibit viral translation and to promote the accumulation of the viral genome that is degraded by the Xrn-1 nuclease to generate more sfRNAs (Schneider and Wolfinger, 2019). In some YFV vaccine strains, there is a mutation in this region that alters its folding, but this does not occur in the 17DD vaccine strain, suggesting its participation in virulence (Proutski et al., 1997). There is evidence that in JEV, the SLII stem–loop acts as a promoter for transcription of sfRNA in an NS5-dependent manner (Chen et al., 2018), and mutations in SLIV favoured viral replication in BHK-21 cells but attenuated neural invasiveness and pathogenicity (Xing et al., 2021). In TBE viruses, alterations of SL3 and SL4 were found to be associated with an increase in virulence in the brain but a reduction in virus multiplication in the periphery in a mouse model (Sakai et al., 2015). These structures were found to be absent in a virulent TBE strain, suggesting that they could act as a spacer separating the folded 3’UTR from the rest of the genome, facilitating the binding of the viral polymerase and cellular factors involved in replication (Muto et al., 2018). SL3, SL4 and SL5 are targets of cellular proteins, such as cold shock domain containing-E1 (CSDE1), interleukin enhancer binding factor 3 (ILF3), spermatid perinuclear RNA binding protein (STRBP) and fragile X mental retardation protein (FMRP). These proteins are involved in processes, such as translation, neuronal development, viral replication, and mRNA translocation. In particular, silencing FMRP reduces TBE replication in SH-SY5Y cells (Muto et al., 2018).

The core region is characterized by two almost identical “dumbbell” structures, designated DB1 and DB2; an A-rich region (AR1), which contains a putative cyclization sequence; and a short hairpin (A2). In DENV, the two dumbbell structures are flanked by A-rich sequences (AR1-3). A five-base motif within AR1 and AR2 can base pair to nucleotides in the loops of DB1 and DB2, leading to the formation of the pseudoknots PK1 and PK2. This basic structure is also observed in JEV, WNV, MVE and KUN. The DB2 structure is absent in YFV and ZIKV, and both DB1 and DB2 are absent in the TBE genome; however, the formation of pseudoknots is apparently a common feature observed in all MBFV (Olsthoorn and Bol, 2001; Funk et al., 2010; Kasprzak and Shapiro, 2014; de Borba et al., 2019; Dayananda et al., 2021). These structures are relevant for replication efficiency, since mutations that disrupt them result in the generation of DENV-3 with an attenuated phenotype in SCID-Huh-7 mice, mosquitoes, and rhesus monkeys (Blaney et al., 2008; de Borba et al., 2019) and a reduction in the replication efficiency of KUN (Funk et al., 2010). The DB structures of DENV-2 interact with DDX6, a protein that has pro-viral activity in the assembly or release of viral particles (Ward et al., 2011). They are also structures that interrupt the activity of Xrn-1 nuclease to generate some populations of sfRNAs by mosquito-adapted DENV (Filomatori et al., 2017) and KUN (Funk et al., 2010). In ZIKV, there is a putative binding site for the Msi protein (Schneider and Wolfinger, 2019). DB1 and its pseudoknot in WNV (Berzal-Herranz et al., 2022) and DB2 in duck Tembusu virus (DTMUV) (Wang et al., 2020) are particularly important in viral translation. In JEV, mutations in DB1 promote viral replication in BHK-21 cells but reduce the neuroinvasiveness and neuropathogenicity of the virus (Xing et al., 2021). Finally, associated with the DB structures (Funk et al., 2010; Kasprzak and Shapiro, 2014), there are two putative cyclization sequences denoted RCS2 and CS2 that participate in viral translation enhancement in DENV (Wei et al., 2009).

The 3’-terminal regions of MVE, YF and DENV viruses have a cyclization sequence (CS1), a small hairpin (sHP), and a 3’ hairpin structure from 87 to 96 nucleotides (Hahn et al., 1987; Villordo et al., 2010).

CS1 is a sequence of 26 nucleotides complementary to others present in the 5’ UTR. The interactions between the cyclization sequences in the 5’-and 3’-terminal regions result in a conformational change that is a prerequisite for self-primed viral RNA synthesis by the viral RNA polymerase (Hahn et al., 1987; You and Padmanabhan, 1999) and probably for viral translation inhibition (Wei et al., 2009).

Downstream of the CS1 sequence, there is a small region with low nucleotide conservation among MBFV. In contrast, in DENV, the CS1 sequence forms a conserved secondary structure designated sHP, which is important for RNA synthesis but not translation. The 3’ UAR, which interacts with the complementary 5’ UAR present in the 5’ UTR, is extended from the sHP to the 3’ hairpin stem (Villordo et al., 2010).

The 3’ hairpin or 3’SL is located at the very end of the viral genome and is also found in NKVF and ISVF (Ochsenreiter et al., 2019). It has an important function in viral translation and replication. In fact, the YFV vaccine strain has a mutation in this region that alters its folding, suggesting its participation in virulence (Proutski et al., 1997).

The top of the 3’SL structure has been the target for designing synthetic nucleic acid derivatives called peptide nucleic acids (PNA) that inhibit JEV replication in BHK-21 cells (Yoo et al., 2009). It has an apparent dual function in viral translation, since it enhances viral translation independently of the cap structure, a process that could be facilitated by interaction with some cellular proteins (Holden and Harris, 2004), but experiments performed with DENV indicate that two sequences in this region are responsible for viral translation inhibition (Wei et al., 2009). In DENV4, the CS1-SL region of the 3’UTR binds to cellular proteins, such as EF-1α, PTB, and autoantigen La (De Nova-Ocampo et al., 2002; García-Montalvo et al., 2004). The p100 protein interacts specifically with the 3’SL region of DENV-2, probably stabilizing the viral RNA, and the silencing of this protein reduces viral replication (Lei et al., 2011). In ZIKV, there is a putative binding site for the Msi protein (Schneider and Wolfinger, 2019). These proteins might facilitate interactions with other cellular or viral proteins to initiate replication or may function as helicases, chaperones, or trans-acting factors in viral replication (De Nova-Ocampo et al., 2002; Lei et al., 2011).

In WNV, the 3’ SL structure is essential for viral replication but not for viral translation. It has been demonstrated that the conserved penta-nucleotide 5’ CACAG 3’ located in the top of this stem–loop structure is required for viral replication, particularly the nucleotides in the first, second, third and fifth positions (Tilgner et al., 2005).

In the ZIKV genome, at 13 nucleotides from the end of the 3’UTR, a G-quadruplex sequence (PQS) characterized by the presence of two or more contiguous runs of guanosines in a short sequence has been identified. The guanosines fold around potassium cellular ions to form G-tetrads, and they can be used as targets for drugs. These PQSs have been identified in other flaviviruses but within the coding region, not the 3’UTR. These PQSs in RNA molecules have been associated with mRNA splicing, transcriptional termination, and translational control; however, their function in the context of viral genomes is not completely understood. This PQS located at the end of the 3’ end of the ZIKV genome could likely be associated with viral replication (Fleming et al., 2016).

## miRNAs with a target in the flavivirus 3’UTR

Several studies have identified miRNAs that are targets of cellular genes important to flavivirus infections (Su et al., 2021; Polonio and Peron, 2021), but these viruses can also generate noncoding RNAs with important functions in replication and pathogenesis, such as sfRNAs and viral miRNAs (vmiRNAs). Such noncoding RNAs are not the aim of this review; for more information, we suggest to the readers the excellent review by Clarke et al. (2015).

Some miRNAs have been shown to able to interact with the viral genome with repercussions in the viral replicative cycle (**Table 1**). For example, overexpression of miR-548g-3p, an IFNβ-inducible miRNA that interacts with the SLA element present in the 5’ UTR of the dengue virus genome, downregulates viral RNA accumulation and viral protein expression in U937 cells (Wen et al., 2015). MiR-484 and miR-744 have target sequences in the SL element of the 3’ UTR of all four DENV serotypes, and their overexpression suppresses DENV-2 NS1 protein production (Castrillón-Betancur and Urcuqui-Inchima, 2017). Most likely, the binding of these miRNAs with important viral genome elements interferes with the interaction of viral or cellular proteins, such as NS5 or PTB, or prevents genome circularization, which are all events that are important for viral translation and replication (Wen et al., 2015; Castrillón-Betancur and Urcuqui-Inchima, 2017).

**Table 1:** Cellular miRNAs with a target in flavivirus genomes.

miRNA	Virus	Model	Effect	Reference
miR-548g-3p	DENV	293T, BHK-21, and Vero cells	Reduces viral replication and translation	Wen et al., 2015
miR-484	DENV	Vero cells	Reduces viral infection	Castrillón-Bentacur and Urcuqui-Inchima, 2017
miR-744	DENV	Vero cells	Reduces viral infection	Castrillón-Bentacur and Urcuqui-Inchima, 2017

The antiviral effect shared by several miRNAs has been evaluated in more detail. Many studies have analysed the effects of the insertion of miRNA recognition elements (MREs) into the viral genome (**Table 2**). The experiments usually consist of the use of a genetically modified virus with a MRE inserted, frequently in the 3’UTR (Lee et al., 2010; Heiss et al., 2011; Pham et al., 2012; Yen et al., 2013). MREs are selected according to the tropism of the virus; for example, miR-122 is expressed in Huh-7 cells (Lee et al., 2010) and miR-142 is expressed in macrophages and dendritic cells (Pham et al., 2012), and their MREs were inserted into the DENV genome. Let-7c, miR-9, miR-124a, miR-128a, and miR-218 are highly expressed in the brain and were selected to test chimeric tick-borne encephalitis/dengue type 4 virus (TBEV/DENV-4) (Heiss et al., 2011); miR-124 MRE was chosen to be inserted into JEV (Yen et al., 2013) and Langat virus (LGTV), a member of the TBEV group (Tsetsarkin et al., 2017). These insertions resulted in inhibition of replication/translation of these viruses in cell lines (Lee et al., 2010; Heiss et al., 2011; Pham et al., 2012; Yen et al., 2013; Tsetsarkin et al., 2017) or primary cell cultures (Heiss et al., 2011), indicating the efficiency and specificity of these miRNAs. Additionally, animal models have been a useful tool to evaluate these viruses. For example, adult mice were inoculated intracerebrally with TBEV/DENV-4 and did not display any neurological symptoms. In this viral construct, prM and E protein genes of DENV-4 were replaced by the corresponding genes of the neurovirulent but nonneuroinvasive Far Eastern strain of TBEV; these TBEV genes carry the MREs of let-7c, miR-9, and miR-124a. Immunodeficient mice (SCID) were completely resistant to viruses with let-7c and miR-124 MREs after intraperitoneal inoculation. Viruses with miR-9 and miR-128a MREs display a decrease in neuroinvasiveness in this animal model (Heiss et al., 2011). Similar results have been observed in mice inoculated with LGTV virus after insertion of the target sequence of miR-124 and/or miR-9 into the viral genome. In another study, insertion of the MRE for miRNA-142 into the DENV genome reduced viral spread in a mouse model (Pham et al., 2012). JEV carrying the miR-124 MRE displays an attenuated phenotype in mice inoculated by either intraperitoneal or intracerebral routes, and this modified virus replicates deficiently in the brain, where miR-124 is highly expressed, but not in the liver or spleen (Yen et al., 2013).

**Table 2:** miRNA recognition element artificially inserted in flavivirus genomes.

miRNA	Virus	Model	Effect	Reference
miR-122 MRE	DENV	BHK-21 and Huh-7 cells	Inhibition of replicon translation	Lee et al., 2010
mir-9 MRE	TBEV/DEN4	C6/36, Vero, primary neuronal cells, and Swiss mice	Reduction in neurovirulence	Heiss et al., 2011
miR-124 MRE	JEV	ICR mice BHK-21 and N18 cells	Reduction in viral infection and neurovirulence	Yen et al., 2013
miR-124 MRE	TBEV	Vero and ISE6 cells Swiss Webster mice	Reduction in viral titers, neuropathogenesis, and neuroinvasiveness	Tsetsarkin et al., 2017
miR-124a/miR-14/miR-1175 MRE	WNV	Vero and C6/36 cells CD-1 mice	Attenuation and protection against viral challenge	Brostoff et al., 2016
mir-124a MRE	TBEV/DEN4	C6/36, Vero, and primary neuronal cells Swiss Webster mice	Reduction in neurovirulence	Heiss et al., 2011 Heiss et al., 2012
mir-128a MRE	TBEV/DEN4	C6/36, Vero, and primary neuronal cells Swiss Webster mice	Reduction in neurovirulence	Heiss et al., 2011 Heiss et al., 2012
let-7c MRE	TBEV/DEN4	C6/36, Vero, and primary neuronal cells Swiss Webster mice	Reduction in neurovirulence	Heiss et al., 2011 Heiss et al., 2012
miR-142 MRE	DENV	Hematopoietic cells Human fibroblasts BHK-21 and HEK293 cells	Reduction in viral translation	Pham et al., 2012

The above experiments have been useful for studying the influence of miRNAs on flavivirus genomes, but they all employed artificial flaviviruses. Another strategy proposed to control flavivirus infections is the design of miRNAs using the viral genome as a template (**Table 3**). These artificial miRNAs (amiRNAs) have been designed against conserved regions of DENV-2 or JEV genomes, cloned into an expression vector, and transfected into susceptible cell lines. These amiRNAs, especially those directed against the 5’CS and E protein coding regions, have been shown to inhibit DENV infection in BHK-21 and Huh7 cells (Xie et al., 2013). Similar results have been obtained using amiRNAs targeted to the 3’ UTR of JEV and N2a neuronal cells (Sharma et al., 2018).

**Table 3:** Artificial miRNAs against flavivirus genomes.

miRNA	Virus	Model	Effect	Reference
amiRNAs	DENV	BHK-21 and Huh7 cells	Inhibition of viral replication	Xie et al., 2013
amiRNAs	JEV	HEK293T and N2a cells	Inhibition of viral infection	Sharma et al., 2018

## Bioinformatics predictions: Finding the best algorithm for miRNA-3´UTR flavivirus genome interactions

There are two main methods to identify miRNAs that have targets in the flavivirus genome. The first is the use of *in vitro* or *in vivo* strategies (Girardi et al., 2018), which can be costly and time-consuming. The second is to perform bioinformatics analysis to increase the possibility of defining a panel of miRNAs that target the flavivirus genome. A large set of miRNA target prediction tools have been developed, including databases, such as miRSystem, mirPath, STarMir, miRDB, and PicTar, and target prediction software programs, such as TargetScan, miRmap, and RNAhybrid. In most circumstances, prediction software has more flexibility to predict a large group of mRNA targets and miRNAs. All these programs are based on conserved seed complementarity to the 3’UTR of host-coding mRNAs, and this strategy has been implemented as the gold standard for *in vitro* prediction of candidates in a short period of time (Peterson et al., 2014). However, since they can show a large number of false-positives, the results obtained using these programs must be considered with caution. Furthermore, not all potential targets are necessarily assessed by the same programs, making it difficult to find a consensus target. Furthermore, although the algorithms are based on target prediction using interspecies conservation of binding sites of miRNAs that were maintained during evolution (Girardi et al., 2018), not all species are recorded. This is the case for prediction targets in mosquitoes; a very small number of algorithms were considered for invertebrates, including RNAhybrid (Krüger and Rehmsmeier, 2006) and TargetScanFly (Wessels et al., 2019). It is well known that the processing of miRNAs in insects is different from that in mammalian cells (Asgari, 2013).

Another limitation of miRNA target algorithms is the existence of non-canonical miRNA-mRNA interactions that can extend the number of potential targets, and even do not include information on miRNA or mRNA expression levels, casting doubt on the real relevance of these targets (Girardi et al., 2018) even in viral infections models. However, some works have been developed to predict the cellular miRNAs targeting viral genomes. ViTa is a database that compiles all available information of host miRNAs, including humans, mice, rats, and chickens, that have target sites on viruses. Also, the database includes viral miRNAs that potentially bind to host mRNAs (Hsu et al., 2007). MirTarP tool was designed to identify 2557 possible human miRNAs to have targets in viral genomes (3133 viral genes and 3376 viral proteins) of 34 families, including seven different genome types: ssDNA, dsDNA, no RNA stage, dsRNA, ssRNA, retroviruses, deltaviruses, and unclassified viruses (Shu et al., 2018).

Other computational studies have suggested that endogenous miRNAs can potentially interact with non-coding regions of flavivirus genomes. For example, using ViennaRNA and miRanda algorithms, it has been demonstrated that the genome of the four DENV serotypes shows targets for human miRNAs independently and exclusive for each serotype, suggesting that the structure and nucleotide composition of the viral genome is important to develop applications. Using this approach, 52 miRNAs were exclusively identified for DENV-1 and 3, 47 for DENV-2, and 20 for DENV-4. In addition, miR-548g-3p, miR-6828-3p, miR-4692, miR-1914-3p, and miR-3191-5p were found to target all DENV serotypes (Valadares et al., 2018). Baig and Krishnan identified a total of 30 tissue-specific human miRNAs in cells with hematopoietic origin that could bind to the 3’UTR of all four DENV serotypes using four target predictor algorithms, StarMir, RNA22, viz.miRanda, and RNAhybrid. These studies suggest the possibility that more than one miRNA can bind to 3’ UTR of all DENV genomes including the miR-6824–3p, miR-4787–5p, miR-615–5p, miR-6787–5p, and miR-661 (Baig and Krishnan, 2021). On the other hand, some miRNAs have been identified by computational analysis that can bind in more than one region of the flavivirus genome, including the mosquito miR-980 and miR-263 which can potentially target more than one location in ZIKV genome (Saldaña et al., 2017), and the human miRNAs miR-6787–5p and miR-615–5p in DENV (Baig and Krishnan, 2021).

Despite the limitations in this field of research, several works have been conducted using bioinformatics analysis to understand the targets of miRNAs on the 3’UTR of the flavivirus genome, and some of them have been validated by *in vivo* assays, including remarkable works from Castrillón-Betancur and Urcuqui-Inchima where they use two algorithms predictors, MicroInspector and RNAhybrid, and found that miR-484 and 744 bind to the 3´UTR of all four DENV serotypes. The computational results were validated using the GFP reporter gene fused to the 3′ UTR of DENV and the results showed that this interaction decreased viral replication (Castrillón-Betancur and Urcuqui-Inchima, 2017). In other studies, bioinformatics analysis using MicroInspector and RNAhybrid showed similar results for miR-133a which targets the 3´UTR of DENV, and its overexpression in Vero cells reduces the viral replication. Again, the miR-133a targeting was validated using GFP reporter gene (Castillo et al., 2016).

**Figure 2:**
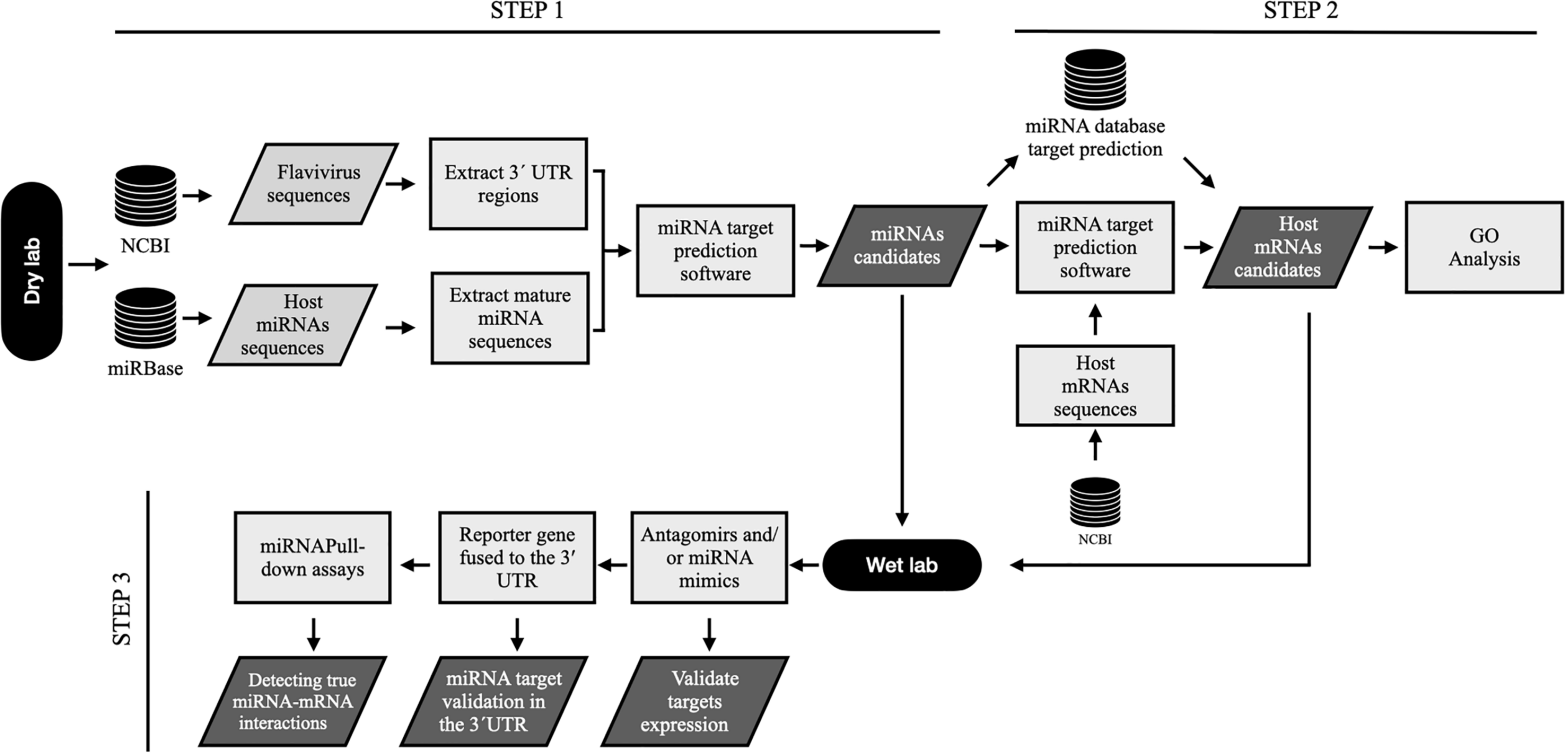
. Workflow to predict microRNA targets within genomic 3´UTR sequences of flavivirus and their relevance to biological processes. Step 1, flavivirus genome sequences and host miRNA are selected from NCBI and miRBase databases, respectively. Then, it is necessary to extract the full-length flavivirus 3’UTR sequences as well as the host microRNA sequences to predict all potential miRNA targets using algorithms predictors. Step 2, after the selection of miRNA candidates, it is necessary to determine the cellular mRNA targets to demonstrate the possible biological relevance of these microRNAs. For this, host mRNA sequences are extracted from NCBI, and target prediction is performed for the selected miRNAs. Similarly, mRNA targets can be found using databases and previously reported studies. All mRNA candidates can be used for GO analysis to determine the biological relevance of these miRNAs. Step 3, finally, miRNA candidates and targets are validated in the laboratory. *In vitro* validations can be performed using functional analysis such as transfection of inhibitors (antagomirs) or miRNA mimics, followed by target mRNA expression (qPCR or RNAseq) and protein expression (Western blotting or ELISA). Reporter gene assays, including luciferase and GFP assays, are excellent tools to demonstrate miRNA-3´UTR interactions. Biotinylated miRNA are useful probes for the identification of miRNA:mRNA interactions. For this, miRNA can be marked with biotin and transfected into the cells; then, the miRNA is sequestered and recovered (pull-down) with all possible RNA and protein interactions. The 3´ UTR can be detected in the pull-down assays using qPCR (CLIP-qPCR) o RNAseq (CLIP-Seq).

In conclusion, bioinformatic analysis is a good tool to reduce time and process in miRNA target identification. However, all algorithms must be experimentally evaluated to identify the proposed results with greater efficiency and reality. In our opinion, to assume a correct identification of the targets in the 3’UTR region of flaviviruses, it would be necessary to carry out a workflow that leads from the prediction of targets of miRNAs to their validation using in experimental assays (**Figure 2**).

## Potential clinical applications of miRNAs:

Drugs specifically approved as anti-flavivirus drugs are scarce (Zakaria et al., 2018; Zhao et al., 2021), the number of licensed vaccines against flaviviruses is limited, and many of them are in a state of development. Even the vaccines against YFV and JEV have been effective, others have problems such as secondary effects, low effectiveness, and high cost (Araujo et al., 2020; Wollner and Richner, 2021; Zhao et al., 2021).

DENV virus represent a special problem in vaccine development. There is a licensed vaccine named Dengvaxia^®^ by Sanofi-Pasteur laboratories consisting in a YFV backbone where the prM and E sequences were replaced by the corresponding of the four DENV serotypes, and it is only approved to be used in 9-16 year-old individuals with previous infection with DENV because it does not induce an equivalent immune response against all serotypes, particularly against DENV-2. Its application in naïve children has been associated with severe dengue illness (Araujo et al., 2020; Shukla et al., 2020; Halstead, 2021). During primary DENV infections, the antibodies generated are sufficient to neutralize the virions of that specific serotype. However, during a second contact with the virus, especially with a different serotype, the antibodies induced during the first infection are not able to neutralize the virus. Moreover, these antibodies facilitate the infection to dendritic cells and monocyte/macrophages through the Fc receptor and modify the cellular environment to facilitate viral replication including inhibition of interferon signaling, nitric oxide synthesis, and RIG-1 and MDA-5 genes, and upregulation of IL-10 production and autophagy (Khandia et al., 2018). This phenomenon is called Antibody Dependent Enhancement (ADE) and it is strongly associated with severe dengue outcome (Ulrich et al., 2020; Halstead, 2021). It has been proved that the antibodies raise against domain III of DENV E protein have a high neutralizing activity (protective antibodies) but those raise against prM and the fusion loop of E proteins are pathogenic antibodies that promote ADE (Shukla et al., 2020). Additionally, it has been reported that antibodies generated during ZIKV infection could cross-react with DENV and induce ADE (Martín-Acebes et al., 2018).

There are several types of vaccines. The live attenuated vaccines, like anti-YFV and anti-JEV, are usually very effective but they can cause serious side effects after vaccination and there is the risk of the appearance of revertant viruses. On the other hand, inactivated vaccines, like anti-TBEV vaccine, have a relatively low immune effect and the immune response time is short. Many of these problems can be overcome using molecularly engineered vaccines (Zhao et al., 2021) and genetically engineered flaviviruses with MREs of miRNAs have a promising future. Immunization of mice with JEV carrying the miR-124 MRE resulted in full protective immunity against subsequent JEV lethal challenge (Yen et al., 2013). Rhesus monkeys inoculated with TBEV/DENV4 chimaeras carrying miR-124 or miR-9 MREs developed high levels of TBEV-specific neutralizing antibodies (Heiss et al., 2011). However, although all these results suggest the use of these engineered viruses as potential vaccine candidates, some problems have been detected. Some mice inoculated with TBEV/DENV-4 chimaeras carrying the MRE of miR-9 or miR-128a died. The viruses recovered from these animals displayed a single nucleotide mutation within the MRE. This mutation allowed the virus to escape miRNA-mediated inhibition (Heiss et al., 2011). Similar results were observed with the same chimeric virus with the MRE of miR-124a. In this case, single- or double-nucleotide substitutions in the central part of the miR-124a MRE or partial or complete deletion of this element were detected. The inoculation of mice with a high dose of JEV targeted to miR-124 also resulted in mouse death, and the recovered viruses displayed a deletion of the MRE of miR-124 (Yen et al., 2013). This problem was solved by inserting several copies (two to four) of the MRE of miR-124 into the TBEV/DENV-4 virus. The immunodeficient mice inoculated with these viruses did not develop any symptoms of encephalitis (Heiss et al., 2012). In another study, WNV vaccine candidates were developed in a similar manner: insertion of several MREs of miR-124a with perfect complementarity in tandem flanking regions of the UTR necessary for efficient viral replication. These vaccines were capable of eliciting a neutralizing antibody response in mice, and two constructs provided 100% protection (Brostoff et al., 2016). All these studies provide good evidence that the miRNAs could be a useful tools in the development of vaccines against almost all flaviviruses; however, the problem of the ADE phenomenon still persist in the case of DENV and ZIKV. The better vaccine to prevent DENV infection should induce a strong humoral and cellular immune response against all four serotypes but specially antibodies against viral epitopes that do not facilitate ADE, like the domain III of E protein (Shukla et al., 2020). The insertion of MREs in the 3’UTR of DENV genome would result in an attenuated virus but still able to generate antibodies that could cause ADE. One strategy to solve this problem is using miRNAs directly as anti-viral agents. Although there are not studies in flaviviruses using animal models, the antiviral therapy against viruses using miRNAs has been tested. For example, adeno associated vectors (AAV) expressing amiRNAs targeting against preterminal (pTP) and E1A viral proteins reduced viral titers, genome copy number and liver injury in immunosuppressed Syrian hamsters infected with human adenovirus 5 (hAd5) (Schaar et al., 2017). A similar strategy has been used in hepatitis B virus (HBV). AmiRNAs targeting the S and C regions of the genome of this virus were expressed using AAV8 vectors and a liver-specific promoter. They were inoculated in transgenic mice 2F, a persistent and highly replicating HBV model. These mice showed a reduction in the serum levels of HBsAg and HBeAg; and in viral particle DNA, viral RNA copy number, and presence of HBcAg in the liver in a dose dependent manner. Additionally, those vectors were tested in other two lines of transgenic mice, one middle (C16) and one high (C2) HBV replicative models with similar results (Mao et al., 2022).

In other study, a combination of 5 cholesterylated stable and modified miRNA (agomirs) was prepared in saline buffer. It included hsa-mir-127-3p, hsa-mir-486-5p, hsa-mir-593-5p, and mmu-mir-487b-5p with targets in the 8 genome segments of PR8 (H1N1) influenza virus, and hsa-miR-1-3p with targets in the host ATP6V1A, a protein that regulates viral replication. Intranasal administration of this combination to BALB/c mice resulted in a moderate weight loss and reduction in lung damage after influenza virus infection with reduction in viral mRNA and protein levels (Peng et al., 2018). Finally, locked nucleic acid (LNA) oligonucleotides complementary to miR-K1, miR-K4 and miR-K11, all miRNAs synthetized by Kaposi’s sarcoma-associated herpesvirus (KSHV) and involved in cellular proliferation and survival, were linked to carbon dots acting as a delivery agents. The intraperitoneal inoculation of these LNA associated to carbon dots in a xenograft Nod/Scid mouse model of primary effusion lymphoma (PEL), was effective in preventing the establishment, growth and spread of the lymphoma apparently by their capability to induce apoptosis (Ju et al., 2020). Together, all these reports propose a specific antiviral strategy that can be used against flaviviral infections in the future.

## Concluding remarks

Flaviviruses are important pathological agents that cause important diseases in humans, from mild fever to encephalitis and haemorrhagic fever, which in many cases are potentially fatal. There are no antiviral drugs to treat diseases caused by flaviviruses, and the number of vaccines to prevent them is still limited. miRNAs are small noncoding RNAs that are able to control eukaryotic gene expression at a posttranscriptional level, but recently, those miRNAs have been discovered to also be capable of interacting specifically with viral genomes and to have either pro- or anti-viral effects. In the case of flaviviruses, almost all miRNas that interact with the viral genome have an inhibitory effect. In fact, this property of some miRNAs has been exploited to design engineered viruses for vaccination purposes; however, although experimental results have been promising, these experiments have only been performed in animal models and with a limited number of viruses. Bioinformatic software is a useful tool that combines a large amount of data and performs predictions that can then be applied to experimental procedures. In fact, the available information on flaviviral genome sequences and miRNAs showed that there are many miRNAs able to interact with the genome of more than one flavivirus, and humans display a higher number than mosquitoes. The miRNAs identified here should be tested *in vitro* and *in vivo*, and this information will be useful to design antiviral therapies in humans or even strategies directed at the vector to reduce transmission. In this way, miRNAs have a promising future as therapeutic molecules.

## Author contributions

RA-B: conceptualization, investigation, and writing. JS-B: conceptualization, investigation, and writing. All authors contributed to the article and approved the submitted version.

## Funding

This work was supported by Secretaria de Investigación y Posgrado of Instituto Politécnico Nacional (Project SIP 20221502). Dr. JS-B has a fellowship from Estímulo al Desempeño de los Investigadores (EDI) and Comisión de Operación y Fomento a las Actividades Académicas (COFAA) of Instituto Politécnico Nacional. Dr. RA-B and JS-B have fellowships from Sistema Nacional de Investigadores of Consejo Nacional de Ciencia y Tecnología (CONACyT), Mexico.

## Conflict of interest

The authors declare that the research was conducted in the absence of any commercial or financial relationships that could be construed as a potential conflict of interest.

## Publisher’s note

All claims expressed in this article are solely those of the authors and do not necessarily represent those of their affiliated organizations, or those of the publisher, the editors and the reviewers. Any product that may be evaluated in this article, or claim that may be made by its manufacturer, is not guaranteed or endorsed by the publisher.
